# Stereochemical Trajectories of a Two-Component Regulatory System PmrA/B in a Colistin-Resistant *Acinetobacter baumannii *Clinical Isolate

**DOI:** 10.52547/ibj.25.3.193

**Published:** 2021-03-03

**Authors:** Mohammad Reza Kandehkar Ghahraman, Hossein Hosseini-Nave, Omid Azizi, Mohammad Reza Shakibaie, Hamid Reza Mollaie, Samane Shakibaie

**Affiliations:** 1 Department of Microbiology and Virology, Kerman University of Medical Sciences, Kerman, Iran;; 2 Research Center for Tropical and Infectious Diseases Kerman University of Medical Sciences, Kerman, Iran;; 3 Department of Laboratory Sciences, School of Paramedical Sciences, Torbat Heydariyeh University of Medical Sciences, Torbat Heydariyeh, Iran;; 4 Student Research Committee, Kerman University of Medical Sciences, Kerman, Iran

**Keywords:** Acinetobacter baumannii, Amino acid substitution, Colistin, Mutation

## Abstract

**Background:**

There is limited information on the 3D prediction and modeling of the colistin resistance-associated proteins PmrA/B TCS in *Acinetobacter baumannii*. We aimed to evaluate the stereochemical structure and domain characterization of PmrA/B in an *A. baumannii* isolate resistant to high-level colistin, using bioinformatics tools.

**Methods:**

The species of the isolate and its susceptibility to colistin were confirmed by PCR-sequencing and MIC assay, respectively. For 3D prediction of the PmrA/B, we used 16 template models with the highest quality (e-value <1 × 10−50).

**Results:**

Prediction of the PmrA structure revealed a monomeric non-redundant protein consisting of 28 α-helices and 22 β-sheets. The PmrA DNA-binding motif displayed three antiparallel α-helices, followed by three β-sheets, and was bond to the major groove of DNA by intermolecular van der Waals bonds through amino acids Lys, Asp, His, and Arg, respectively. Superimposition of the deduced PmrA 3D structure with the closely related PmrA protein model (GenBank no. WP_071210493.1) revealed no distortion in conformation, due to Glu→Lys substitution at position 218. Similarly, the PmrB protein structure displayed 24 α-helices and 13 β-sheets. In our case, His251 acted as a phosphate receptor in the HisKA domain. The amino acid substitutions were mainly observed at the putative N-terminus region of the protein. Furthermore, two substitutions (Lys21→Ser and Ser28→Arg) in the transmembrane domain were detected.

**Conclusion:**

TheDNA-binding motif of PmrA is highly conserved, though the N-terminal fragment of PmrB showed a high rate of base substitutions. This research provides valuable insights into the mechanism of colistin resistance in *A. baumannii*.

## INTRODUCTION

*Acinetobacter baumannii *is characteristically surrounded by an outer membrane, which typically consists of phospholipids in the inner leaflet and the LOS in the outer membrane region^[^^1^^]^. The hydrophilic outer region of asymmetric bilayer combined with the lipid A serves as an important permeability barrier to hydrophobic compounds including antibiotics^[^^2^^]^. Interestingly, the LPS/LOS of *A. baumannii* represents not a static structure; it can be modified in various ways when exposed to preventive antibiotics^[^^3^^]^. Nevertheless, the ability to modify the LPS in response to antibiotics like colistin is critical for resistance to this bactericidal agent^[^^1^^,^^2^^]^. Many of these alterations are caused by PmrA/B TCS, which remains a major regulator in chemical modification of LPS. Besides, lipid A, a constituent of the outer membrane, is the prime target of colistin. Colistin-resistant strains of *A. baumannii* have the ability to adversely modify or inevitably lose LOS from the outer membrane due to specific mutations in the* pmr*A/B genes^[^^4^^]^.

 Mechanism of colistin resistance due to mutations in the PmrA and PmrB was first identified in Salmonella enterica serovar Typhimurium mutants that displayed high MIC to polymyxin B^[^^5^^]^. Later, genetic mapping and DNA sequence analysis revealed that the* pmr*CAB operon typically contains PmrC-encoding sequence, which acts in LPS modification. However, phosphorylated PmrA is required for PmrC overexpression^[^^6^^,^^7^^]^. Colistin- resistance in *A. baumannii* requires three genetic steps, including (i) amino acid substitutions in *pmr*B, (ii) overexpression of *pmr*A/B, and (iii) activation of *pmr*C gene^[^^8^^]^. The phosphoethanolamine transferase enzyme, which is encoded by *pmr*C gene, contributes positively to colistin -resistance by adding phosphoethanolamine to the lipid A^[^^5^^]^. This process reduces the specific binding of positively charged colistin to the bacterial outer membrane, impeding the penetration of colistin into the cell^[^^6^^,^^7^^]^. Besides, different numbers, types, and positions of mutations in *pmr*B gene can cause different levels of MIC against colistin^[^^9^^]^; however, not all substitutions could alter bacterial susceptibility to colistin, and the mechanism is still unclear^[^^10^^]^.  Nevertheless, the phosphate transfers to the conserve asparagine residue of the phosphate receptor domain of PmrA protein and causes an adaptive response, such as increase in the transcription of the *pmr*C gene^[^^11^^,^^12^^]^. Currently, the *pmr*B gene seems to be more prone to bacterial mutations compared to the other response regulator *pmr*A. It has been reported that 71% of mutations have occurred in the HAMP and DHP domains^[^^13^^,^^14^^]^. Further sequence analysis of 16 polymyxin B-resistant *A. baumannii* strains, including six spontaneous mutants derived from strain ATCC 17978 and 10 clinical isolates from diverse sources, showed independent mutations in *pmr*B or *pmr*A genes^[^^15^^]^. Moreover, mutations in *pmr*B gene displayed a decrease in the polymyxin B susceptibility in two mutants and two clinical isolates^[^^15^^]^. It was interesting that the PmrB in Pseudomonas aeruginosa, Klebsiella pneumoniae, and A. baumannii showed 29% similarity at amino acid level, while 43% similarity was observed for PmrA in these isolates^[^^12^^]^. 

 The present study aimed to investigate the stereochemical structures, domain characterizations, and amino acid substitutions in the PmrA/B proteins in an *A. baumannii* clinical strain highly resistant to colistin by using different bioinformatics tools.

## MATERIALS AND METHODS

**Bacterial identification and molecular typing**

In our previous study, a colistin-resistant *A. baumannii *was isolated from intensive care unit of a teaching hospital in Kerman, Iran. The isolate was confirmed phenotypically and genetically by biochemical and PCR-sequencing tests as described before^[^^16^^]^. The MIC of colistin-resistant isolate was determined by microbroth dilution test as described by CLSI 2019 (reviewed in^[^^16^^]^). In order to find out the sequence type of the isolate, we performed MLST as per Oxford’s scheme^[^^16^^]^.

**DNA sequencing and gene annotation**

Both strands of *pmr*A/B genes were sequenced by the ABI 373 Prism DNA sequencer (Applied Biosystems 373/3730XL model, Bioneer, Korea)^[^^16^^]^. The nucleotide and amino acid sequences were then compared with known sequences deposited in the NCBI website (https://www.ncbi.nlm.nih.gov) using the BLAST program (www.ncbi.nlm.nih.gov/blast). Predicted proteins were searched against the NCBI nonredundant database. Both the *pmr*A and *pmr*B genes were annotated by using NCBI Prokaryotic Genes Annotation Pipeline software with homology-based methods (https://www.ncbi.nlm.nih.gov/genome/ annotation_prok).

**Nucleotide and protein sequence accession numbers**

Full-length PmrA/B DNA sequences obtained from our previous sequencing^[^^16^^]^ were deposited in the GenBank database (NCBI) under accession numbers MN787072 (*pmr*A) and MN787073 (*pmr*B). The PmrA/B amino acid sequences were also deposited in the NCBI under accession numbers QIC34671 (PmrA) and QIC34672 for (PmrB).

**Prediction of PmrA/B 3D structures and protein simulation**

For the prediction of PmrA/B 3D structures, including the numbers of α-helices and β-sheets, we applied the comparative modeling method using the Expasy SWISS-MODEL Workspace Server (http:// swissmodel.expasy.org/workspace)^[^^17^^]^. Templates were blasted against the primary amino acid sequences of the PmrA/B. The configuration of domains were alignments with similar sequences derived from the protein sequences secondary database of SWISS-Prot^[^^18^^,^^19^^]^. The model was generated based on the target-template alignment using PROMOD software version 3, an in-house comparative modeling engine based on the open structure for each identified template^[^^20^^]^. In the software, pairwise comparison of PmrA/B predicted sequences and structures were matched against a library of 3D profiles (https://pypi.org/project/promod). The templates with the highest quality were then selected for model creation. Furthermore, model quality assessment, including global and local qualities, and Ramachandran clash score were estimated as previously described^[^^17^^]^. Molecular modeling of PmrB and PmrA were also predicted by PSIPRED online software version 4^[^^21^^]^. In the end, positions of α-helices and β-sheets in both the PmrA and PmrB proteins were confirmed by the Protein Homology/AnalogY (Phyre2) website (http://www.sbg.bio.ic.ac.uk/phyre2/html).

**Domain analysis of PmrA/B proteins**

For domain analysis, we carried out multiple amino acid sequence alignments of PmrA/B with those closely related sequences deposited in the UniProtKB online database (www.uniprot.org). The functionally important sites in the signal receiver and the DNA-binding motif of PmrA were determined by the PyMOL visualization program (https://sourceforge.net/ p/pymol/code/HEAD/tree/trunk/pymol/) and HMMs. On the basis of homology to the amino acid sequences of known proteins, putative functions were assigned. To predict the structures of domains and amino acids involved, we used comparative homology modeling, which consisted of four main steps: (1) identification of structures and selection of templates, (2) alignment of the target sequence with the chosen model structure, (3) generation of models for the target structure using information about the structure of the template, and (4) validation of the models generated for the target^[^^22^^]^. It should be mentioned that an overall classification based on the N-terminal sensor kinase domain of PmrB remains problematic since these domains vary greatly in sequence, membrane topology, composition, and domain arrangement that have profound effects on sensing the kinase domain. For these reasons, we predicted the exact positions and arrangements of transmembrane locality, histidine kinase, and HATPase_c domains of PmrB protein by using Pfam software (https://pfam.xfam.org). Superimposition of PmrA protein with that of closely related colistin-sensitive *A. baumannii* strain was performed by MADOKA web server (http://madoka.denglab.org) as described previously^[^^23^^]^. For superimposition, we calculated the e-value, which provides information about the likelihood of a given sequence match obtained. If the e-value is small, the match is significant because it is less likely to be a result of random chance. If e < 1 × 10−50, the database match is most likely to be a result of homologous relationships^[^^24^^]^.

**The evolutionary relationships of domains**

The CATHEDRAL server (http://www.cathdb.info) was used to compare PmrA/B domains against domains already classified in the CATH database version 2.6, which provides a hierarchical classification of domains based on their folding patterns and the number of helices and sheets^[^^25^^]^. We compared PmrA protein in the CATH superfamily and only those sharing 80% of the same members were selected in this study^[^^26^^]^. 

## RESULTS

**Bacterial source**

In our previous study, the MIC value of colistin was determine as 32 µg/mL for the *A. baumannii* isolate 1, and molecular typing by the MLST indicated that the colistin-resistant isolate belonged to a domestic ST type assigned as ST-1752^[^^16^^]^. In the present investigation, we performed the detail stereochemical structures, molecular dynamics, multiple amino acid alignments, and the position of mutations in each domain of PmrA/B two component regulatory system. 

**PmrA structural analysis**

**Fig. 1:**
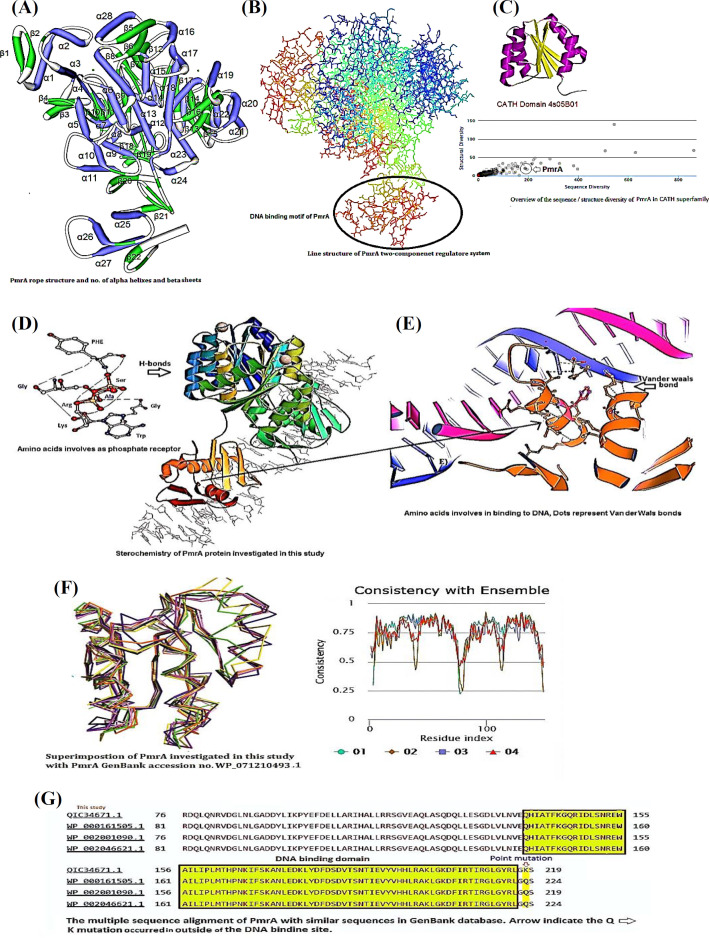
Stereochemical structure and amino acids involved in DNA-binding activity of PmrA in the colistin-resistant *A. baumannii*. (A) PmrA rope structure including the number of α-helices and β-sheets and overall predicted configuration; (B) line illustration including the position of DNA-binding motif; (C) the phosphate receptor domain of PmrA (CATH 4s05B01) and its position on CATH superfamily; (D) cartoon presentation of the activated PmrA dimer in complex with the promoter DNA; (E) structural dynamic of amino acids involved in binding to DNA. The connection was performed via Van der Waals bonds as shown by dots. The Lys, Asp, glycine, Glu, and phenylalanine were representing in DNA-binding sit; (F) superimposition of PmrA was investigated with colistin-sensitive PmrA GenBank (accession number WP_071210493.1); (G) amino acid sequence alignment of PmrA with closely related sequences. The arrow indicates the position of mutation at amino acid 218

The 3D structural analysis of PmrA revealed a monomeric protein containing non-redundant sequences of 24 α-helices and 22 β-sheets (Fig. 1A). Line structure and position of DNA-binding motif are illustrated in Figure 1B. Nevertheless, the other domain acted as a phosphate acceptor belonged to the CATH superfamily, which was designated as 4s05B01 (Fig. 1C). Moreover, CATH analysis indicated that PmrA had close similarities with other PmrA reported in the CATH database. Using highly similar template trajectories, we generated PmrA target displaying the traditional (βα) fold in the DNA-binding domain. It consisted of a central five parallel β-sheet surrounded by three helices. The amino acids involved as phosphate receptors are depicted in Figure 1D. A total of five candidates matched the DNA-binding motif, and we selected the one with the closest similarities. The simulation indicated that the DNA-binding motif was consisted of a unique secondary structure composed of three left-handed of short parallel β- sheets separated by three α-helices in the form of helix-turn-helix DNA-binding topology (Fig. 1D). By Proven (Protein Variation Effect Analyzer: http://provean.jcvi.org/seq_submit.php) software, we found that the intermolecular van der Waals bonds between Ly, Asp, His, and Arg is involved in binding to the major groove of DNA (Fig. 1E). Further superimposing PmrA indicated that, the amino acid substitution Gln→Lys at position 218 occurred just outside of the DNA-binding motif did not affect the DNA-binding activity of PmrA (Fig. 1F and Fig. 1G). Besides, the e-value of the amino acid sequences match was e < 1 × 10−50.

**PmrB structural analysis**

**Fig. 2:**
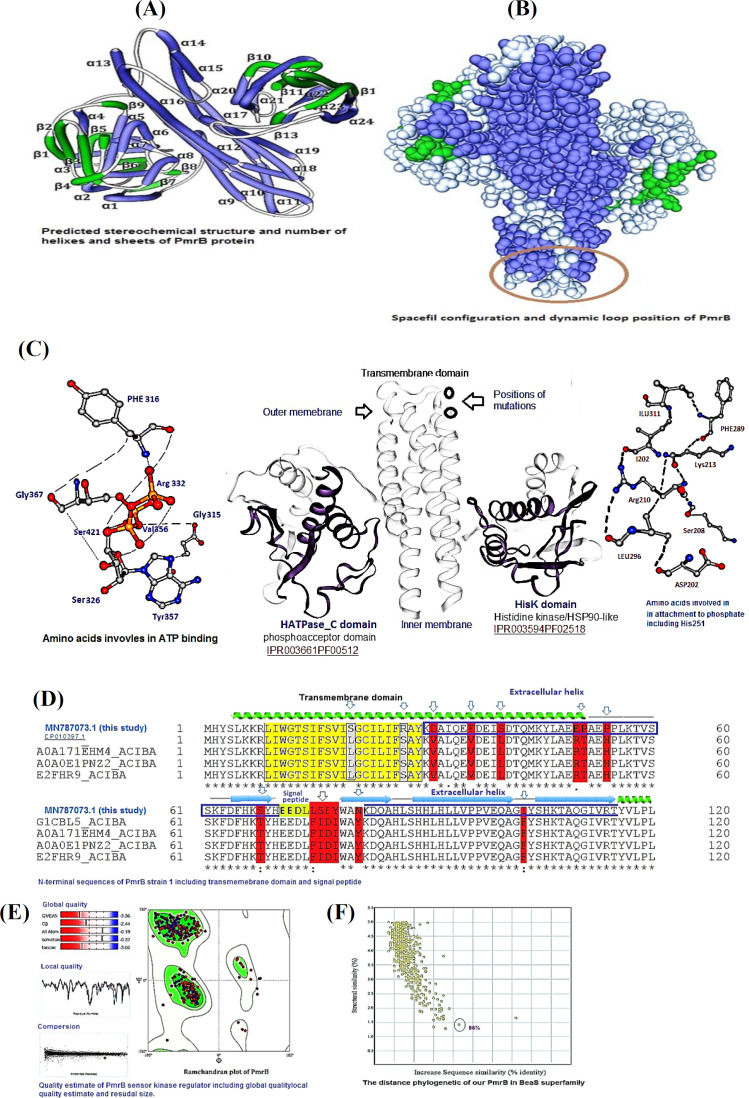
Stereochemical structure of predicted PmrB, positions of domains and analysis of amino acids. (**A)** Rope structure and number of α-helices (blue color) and β-sheets (green color) of PmrB; (**B)** spacefill structure of PmrB and its dynamic D loop position; (**C)** stereochemical trajectory of PmrB comprising of domains and amino acids contents. Mutations in transmembrane domain of PmrB and amino acids residues in HATPase_ c domain are shown with black circles and red color, respectively; (**D)** amino acid substitutions Lys→Ser at position 21 and Ser→Arg at position 28 in the outer region of transmembrane domain of PmrB; (**E)** quality estimate of predicted PmrB sensor kinase. Structure validation by Ramachandran plot depicts the general as well as specific distribution of amino acids. (**F)** Distance sequence relationship and evolutionary similarity of PmrB in this study with superfamily reported so far

**Fig. 3:**
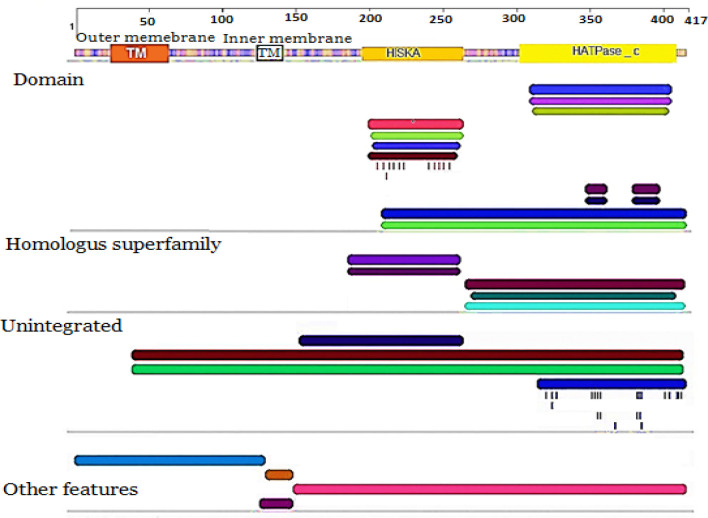
Positions and arrangements of transmembrane, HisKA, and HATPase_c domains on PmrB protein. The analysis was performed by Pfam software

In contrast, PmrB, a monomeric protein, contained 24 α-helices and 13 β-sheets (Fig. 2A). Seven models were analyzed in the Swiss Bioinformatics Resource Portal for the prediction of proper structure and function of PmrB; however, only one model (*A. baumannii* GenBank AEK67613.1) was selected for this study. The PmrB putative dimer was asymmetric containing two chains, A and B. By the Spacefil structure, we found the position of dynamic loop in the PmrB structure (Fig. 2B). This position plays an important role in the transphosphorylation of phosphate group between the domains of PmrB. The catalytic portion of the protein was comprised of a central four α-helices bundle and four β-sheets in antiparallel positions given rise to an orthogonal sandwich structure. In the HisKA domain, the amino acids Ala 224, Ser 214, Gly 216, Ser 215, Tyr 289, Glu 218, Lys 259, and Leu 281, along with His 251 play an important role in phosphorylation and transfer of phosphate group to HATPase-c domain (Fig. 2C). Furthermore, this phosphorylated histidine acted as a phosphate receptor and transferred this phosphate either onto a nucleoside diphosphate or onto a serine residue of the HATPase_c domain. The histidine acceptor site in *A. baumannii* species was highly conserved across the PmrB superfamily, indicating its critical role in kinase function. The transmembrane sensor domain is relatively less conserved. Also, the catalytic HATPase_ c connected to HisKA is illustrated in Figure 3. To gain a representative active conformation, we selected one out of the five trajectories that best fulfilled correct ATP positioning and His-ATP contact distances. Herein, the two putative domains arranged so that the phosphorylatable histidine and ATP became in perfect orientation for phosphoryl group transfer. 

**Evaluation of PmrB domains**

Further analysis of the transmembrane domain of the putative PmrB protein revealed a large twisted antiparallel α-helices (α13 and α14), which vertically traverses the whole domain. The conformation was in form of a helix-turn-helix with outer and inner cytoplasmic subunits (Fig. 2C). Based on sequence and structure comparisons of the HATPase_c domain, we showed that Tyr 357, His 325, Ser 326, Val 356, PHE 316, Gly 367, Gly 315, and Ser 421 could act as a photoreceptor Pribnow box, which binds to the ATP moiety and is required for the autophosphorylation of the PmrC protein. Analysis of amino acid sequence alignment further showed two substitutions in transmembrane domain as Lys→Ser at position 21 and Ser→Arg at position 28 (Fig. 2D). Mutations in transmembrane domain might result in the loss of domain conformation, thereby promoting the phosphorylation of PmrB. Nevertheless, the high ratio of amino acid substitutions was found in the N-terminal region of PmrB where several different amino acid substitutions were detected (Fig. 2D). Perhaps, these mutations, especially Lys to Ser at position 21 and Ser to Arg at position 28, playing a crucial role in the induction of resistance to colistin. The Ramachandran diagram revealed that both the right-handed and the left-handed helices were among allowed conformations (Fig. 2E). Further structural neighborhood analysis of PmrB sequences in this study with closely related sequences in the NCBI database indicated a little evolutionary relationship between our PmrB sequences and those reported previously (Fig. 2F). It should be mentioned that, an overall classification based on the N-terminal sensor kinase domains remains problematic since these domains vary greatly in sequence, membrane topology, composition, and domain arrangement. All of these features have profound effects on sensing and signal transduction to the kinase domain. For these reasons, we predicted the positions and arrangements of transmembrane locality, histidine kinase, and HATPase_c domains of PmrB protein by using Pfam software (Fig. 3).

## DISCUSSION

Currently, there is a paucity of information regarding the detailed stereochemical structure of PmrA/B proteins in colistin-resistant *A. baumannii*. In this investigation, we presented the stereochemical structures, generated molecular dynamics, and key amino acids involved in each domain, including the DNA-binding motif of PmrA and the transmembrane, histidine kinase, and HATPase_c domains of PmrB sensor kinase. We found that His 251 in PmrB and Ser 421 in PmrA are involved in photoreceptor and ATP binding, which binds to the ATP moiety and requires for autophosphorylation of the PmrC protein.

Furthermore, alignments of multiple amino acid sequences revealed that the DNA-binding motif of PmrA was highly conserved across the *A. baumannii* species. This domain forms a symmetric dimer mediated by the 3-α helices and 3-β sheets in antiparallel manner and involves several residues that are highly conserved in the CATH subfamily. Further modeling assessment by superimposing with a colistin-sensitive *A. baumannii* (GenBank accession no. WP_071210493.1) confirmed that Glue→Lys substitution at position 218 in PmrA occurred just outside the DNA-binding motif and did not affect the overall conformation of the protein. In contrast, the detailed stereochemical structure of PmrB revealed different nonsynonymous amino acid substitutions in the N-terminus segment of PmrB. In the transmembrane region of PmrB, two substitutions (Lys→Ser at position 21 and Ser→Arg at position 28) might be responsible for colistin-resistant phenomenon. In this study, we suggested that spontaneous mutations in PmrB rather than the acquisition of pre-existing colistin-resistant strain were responsible for resistance to colistin. In a previous study, it has been reported that the mutations in the *pmr*A/B genes, including *pmrA *Pro 102→Arg, *pmr*B Pro 233→Ser, and *pmr*B Thr 235→Asp were the main sources of resistance to colistin^[^^18^^]^. However, in this investigation, we found two putative HisKA and HATPase_c domains arranged in such a way that, the phosphorylatable histidine and ATP became in perfect orientation for phosphoryl group transfer. Our work was in excellent agreement with the concomitantly published work of Dago *et al.*^[^^27^^]^ who suggested a significant contact between HisKA and α17 HATPase_c. The catalytical region of these proteins consists of a conserved cytoplasmic two-domain core, which features a homodimeric four α-helices harboring the phosphorylation site. Formerly, it has been reported that the HisKA- and ATP-binding domains recognized by the HATPase_c HMM shared by various classes of ATP-binding proteins^[^^28^^]^.

According to the knowledge on the PmrB structure, position 10 is located in the amino-terminal portion, including the cytoplasmic secretion signal (aa 1-13), which is involved in the delivery process of the phosphate into the cell membrane^[^^29^^]^. Nevertheless, it has been suggested that the increased transcription of lipoprotein transporters and lipoproteins in LPS-deficient *A. baumannii* results in outer membrane remodeling that could promote survival without lipid A. Previous mutational analysis of this interface has been indicated that an increase or a decrease in kinase activity represents a physiological change in the conformation of HisKA domain^[^^30^^]^. So far, only a few mutations have been described in *pmr*A locus, and all of them were detected in the phosphate receiver domain including the glutamine 54→serine substitution^[^^31^^]^. However, as the result of HisKA phosphorylation, the phosphate group is transferred to a conserved aspartate residue of the receiver domain of PmrA, which enhances the binding of PmrA to its DNA recognition site and increases the *pmr*C gene transcription^[^^12^^]^. Similarly, in PmrA DNA-binding motif, several residues from the N-terminus of helix α6 and of the C-terminal β-hairpin form H-bonds with phosphate backbone of DNA. For instance, Arg 210 is involved in the binding to the minor groove of the DNA or Asn188 and Asn196, which are inserted into the major grooves of DNA. The residues Thr187, Val192, and His195 are also involved in hydrophobic interactions with DNA bases^[^^12^^]^. Besides, the HAMP linker domain transduces signals from the transmembrane domain to the catalytically active domain by direct interactions; thus, specific conformational changes in the HAMP linker domain can disturb signal transduction, promoting the phosphorylation of the kinase^[^^32^^]^.

Information regarding the mechanism of colistin-resistant *A. baumannii* is rare. In this study, we predicted that the structures and functional sites of two important proteins, PmrA and PmrB, are involved in resistance to colistin in *A. baumannii*. Similarly, we found that the positions and types of amino acids are involved in PmrA/B domains. Besides, using bioinformatics software, we simulated each protein structure and amino acid involved in functional sites. Likewise, we observed several amino acid substitutions in PmrB N-terminal region and two of them influenced the transmembrane configuration. Moreover, the amino acids, as phosphate receptors and donors, are involved in PmrB. DNA-binding motif of PmrA was conserved though CATH superfamily, and an amino acid substitution was detected outside this region. 
